# Viral metagenomics of the gut virome of diarrheal children with Rotavirus A infection

**DOI:** 10.1080/19490976.2023.2234653

**Published:** 2023-07-13

**Authors:** Siwen Bao, Hao Wang, Wang Li, Haisheng Wu, Chunying Lu, Liang Yong, Qing Zhang, Xiang Lu, Min Zhao, Juan Lu, Jia Liu, Chukwudozie Kingsley Ikechukwu, Juan Xu, Ping Ni, Ying Xiong, Wen Zhang, Chenglin Zhou

**Affiliations:** a Clinical Laboratory Center, The Affiliated Taizhou People’s Hospital of Nanjing Medical University, Taizhou, China; b Department of Laboratory Medicine, School of Medicine, Jiangsu University, Zhenjiang, China; c Department of Clinical Laboratory, The Affiliated Huai’an Hospital of Xuzhou Medical University, Huai’an, China; d Qinghai Institute of Endemic Disease Prevention and Control, Xining, China; e Department of Pharmacy, Yancheng Third People’s Hospital, Yancheng, China

**Keywords:** Viral metagenomics, diarrhea, Rotavirus A, gut virome, enteric virus, phage

## Abstract

Diarrhea is a leading cause of morbidity and mortality in children worldwide and represents a major dysbiosis event. Rotavirus has been recognized as a global leading pathogen of diarrhea. This study is aimed at investigating differences in the gut virome between diarrheal children and healthy controls. In 2018, 76 diarrheal fecal samples and 27 healthy fecal samples in Shanghai and 40 diarrheal fecal samples and 19 healthy fecal samples in Taizhou were collected to investigate the composition of the gut virome. Viral metagenomic analyses revealed that the alpha diversity of the diarrheal virome was not significantly different from that of the healthy virome, and the beta diversity had a significant difference between diarrheal and healthy children. The diarrheal virome was mainly dominated by the families *Adenoviridae*, *Astroviridae*, *Caliciviridae*, and *Picornaviridae*. Meanwhile, the healthy virome also contains phages, including *Microviridae* and *Caudovirales*. The high prevalence of diverse enteric viruses in all samples and the little abundance of *Microviridae* and *Caudovirales* in diarrheal groups were identified. The study introduced a general overview of the gut virome in diarrheal children, revealed the compositional differences in the gut viral community compared to healthy controls, and provided a reference for efficient treatments and prevention of virus-infectious diarrhea in children.

## Introduction

Diarrhea, characterized as a symptom that involves loose, watery stools and increased volume and frequent defecation more than three times during the previous 24 h, is a leading cause of morbidity and mortality in children worldwide, especially in developing countries.^1^ There are approximately 1.3 million deaths annually caused by diarrhea, of which nearly half a million deaths are children younger than 5 y.^2^ In low- and middle-income countries, one in 10 deaths in children under 5 y is attributable to diarrhea.^3^ A diverse range of etiologies can lead to diarrhea, including bacteria (*Campylobacter*, *Escherichia coli*, *Salmonella, Shigella*, and *Vibrio cholera*), viruses (adenovirus, astrovirus, norovirus, sapovirus, and rotavirus), and parasites (*Cryptosporidium*, *Entamoeba* and *Giardia*).^1,4^ Diarrhea can induce severe dehydration due to fluid loss and result in electrolyte disturbances, vitamin deficiencies, and malnutrition. As a result, diarrhea increases the risk for life-threatening illness and has long-term impacts on physical and cognitive developmental delays for under-five children.^5,6^ Although diarrhea-related mortality decreased significantly with the global improvement of sanitation, treatment, and immunization during the past 20 y, diarrhea does damage on children and remains a great health challenge.^5^

Rotavirus, a member of the *Reoviridae* family which comprises 11 segments of double-stranded RNA encoding six non-structural proteins (NSP1-NSP6) and six structural proteins (VP1 to VP4, VP6, VP7), has been recognized as a global leading pathogen of diarrhea.^3^ Ten rotavirus species (A to J) have been classified based on the nucleotide sequences and antigenic differences of the VP6 up to now, of which Rotavirus A accounts for more than 90% of infections in humans.^7,8^ Predominantly transmitted through the fecal-oral route, rotavirus infects enterocytes in the small intestine, causes malabsorption, stimulates intestinal secretion, and activates the enteric nervous system leading to diarrhea.^8,9^ As the third leading etiology associated with mortality among children under 5 y old, behind *malaria* and *Streptococcus pneumonia*, rotavirus was responsible for nearly 130,000 deaths per year throughout the world,^10^ and rotavirus infection can occur at any time of the year, especially in winter months.^11^ Rotavirus-related mortality has significantly decreased over time, in part due to the development of the rotavirus vaccination.^7^

The gut microbiome, an integral and essential component of the human body, is primarily composed of bacteria, archaea, viruses, and eukarya inhabiting the gastrointestinal tract.^12^ Homeostasis and symbiotic interactions between the gut microbial community and humans promote the microbiome performing pathogen defense, metabolism, and immunity modulation functions.^13^ The gut microbial community is relatively stable but can change vastly depending on factors such as age, diet, geography, and nutritional status, and dysbiosis in the gut microbiome has been implicated in conditions such as cancer, obesity, diabetes, and Crohn’s disease.^14^ Diarrhea represents a major dysbiosis event with a marked reduction in taxonomic richness and diversity compared with healthy controls.^6,15^ Lower alpha diversity of the gut microbiome was observed in the European adults with diarrhea.^16^ The study by Hao Chung The *et al*. showed an elevation of *Fusobacterium mortiferum*, *Escherichia*, and oral microorganisms and the depletion of *Bifidobacterium pseudocatenulatum* in the diarrheal patients.^15^ Emerging evidence reveals that the virome of the gut microbiome has a profound influence on human physiology via interactions with the gut bacterial community and changing the host gene expression.^17,18^ Nowadays, researches based on sequencing technology mainly focus on the bacteriome. However, bacterial dysbiosis does not appear to be paralleled by primary changes in the gut virome, and changes in bacterial diversity and richness do not explain virome changes.^19^ Study on the bacteriome of diarrhea is mounting, while virome changes remain a mystery, as a result of which, it is necessary to investigate how the composition and abundance of the gut virome alter after diarrhea.

In this study, fecal samples from children with Rotavirus A infection and healthy controls were collected, and viral metagenomic analyses were used to investigate the composition and differences of the gut virome between diarrheal children and healthy controls. Phylogenetic analyses were performed to investigate the genetic diversity of viruses in the human intestine.

## Results

### Overview of the virome

After library construction and next-generation sequencing on the Illumina platform, 32 libraries totally generated 12,936,292 raw reads (11,140–2,017,958 reads per library). The raw reads were assembled *de novo* with each barcode and then compared with the GenBank non-redundant protein database in NCBI using BLASTx. In 32 libraries, a total of 737, 996 viral genomic reads (287–126,919 reads per library) were identified with significant similarity to known eukaryotic viruses. Thirty-two libraries were grouped into four groups according to the health status of sampled persons and sampling sites, including Diarrheal in Shanghai (15 libraries, including MH01-MH15), Healthy in Shanghai (5 libraries, including MH33-MH37), Diarrheal in Taizhou (8 libraries, including MH16-MH22, MH24), and Healthy in Taizhou (4 libraries, including MH28-MH31). The information about each library was shown in Supplementary Table S1.

### Diversity analysis of the gut virome

**Figure 1.**
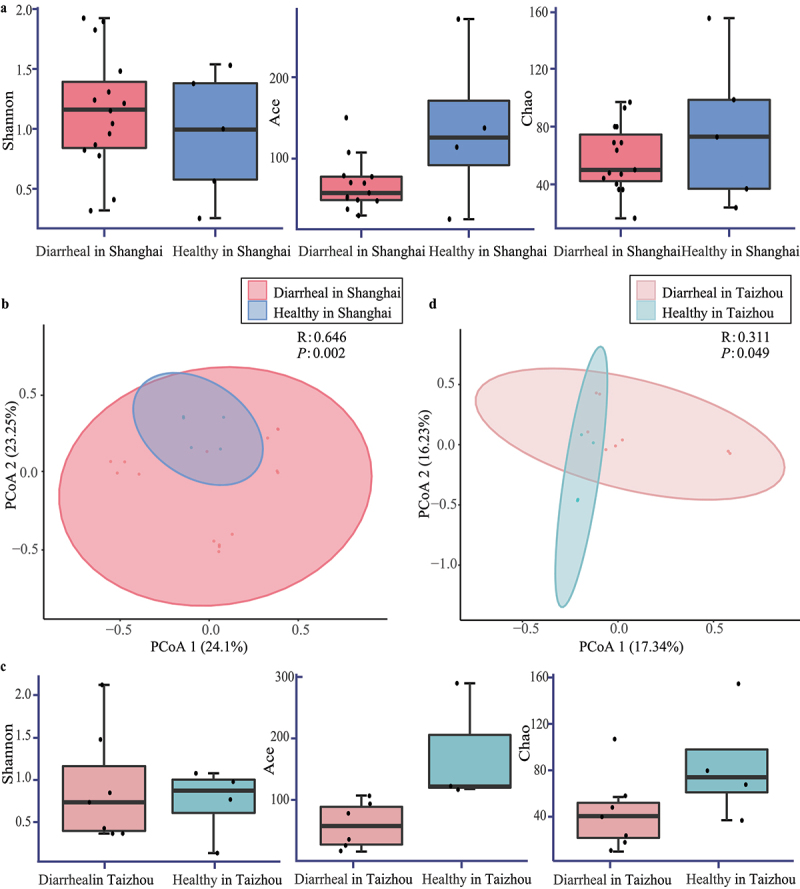
Comparison of viral communities between the diarrheal group and healthy group. Viral reads of the diarrheal groups and healthy groups were calculated and normalized by Megan. (a) Comparison of the alpha diversity was measured with Shannon, ACE and Chao indexes for Diarrheal in Shanghai group and Healthy in Shanghai group using the Wilcoxon test. (b) Comparison of the beta diversity was assessed by unweighted UniFrac distances using PCoA analysis of Diarrheal in Shanghai group and Healthy in Shanghai group. Each plot represents one library and the association between the two groups was measured. (c) Comparison of the alpha diversity was measured with Shannon, ACE and Chao indexes for Diarrheal in Taizhou group and Healthy in Taizhou group using the Wilcoxon test. (d) Comparison of the beta diversity was assessed by unweighted UniFrac distances using PCoA analysis of Diarrheal in Taizhou group and Healthy in Taizhou group. Each plot represents one library and the association between the two groups was measured.

The alpha and beta diversity analyses are essential aspects of microbiological diversity analysis. The alpha diversity analysis is widely used to investigate the richness of species within a community. The beta diversity analysis is often used to assess variations across different communities and to reflect the environmental heterogeneity of species. Viral reads were calculated and normalized to elucidate compositional differences between diarrheal samples and healthy controls. The alpha diversity was assessed by calculating Shannon, ACE, and Chao indexes. The results revealed no significant difference in gut virome of the alpha diversity between group Diarrheal in Shanghai and Healthy in Shanghai but ACE and chao indexes showed that the alpha diversity was slightly lower in group Diarrheal in Shanghai than in group Healthy (Figure 1a). The principal coordinate analysis (PCoA) of the beta diversity was measured by unweighted UniFrac distances and showed that the dispersion of samples in the group Diarrheal in Shanghai was much larger than the healthy control group, which revealed significant differences in beta diversity between group Diarrheal in Shanghai and Healthy in Shanghai (*R* = 0.646, *P* < .01, Figure 1b).

The virome comparison differences between group Diarrheal in Taizhou and group Healthy in Taizhou were illuminated similarly. Alpha diversity did not differ between diarrheal children and healthy controls, which indicated there was no difference in virus diversity in the diarrheal group and healthy group (Figure 1c). PCOA of the diarrheal group and healthy group in Taizhou showed significant differences in beta diversity, which meant comparisons of the gut virome of diarrheal children differed from healthy controls (Figure 1d). The similar results in Shanghai and Taizhou groups showed that the gut virome of diarrheal children caused by Rotavirus infection had no differences in the viral taxonomic diversity compared to healthy controls, while the composition of the viral community differed significantly between diarrheal children and healthy children.

### Composition of the gut virome

**Figure 2.**
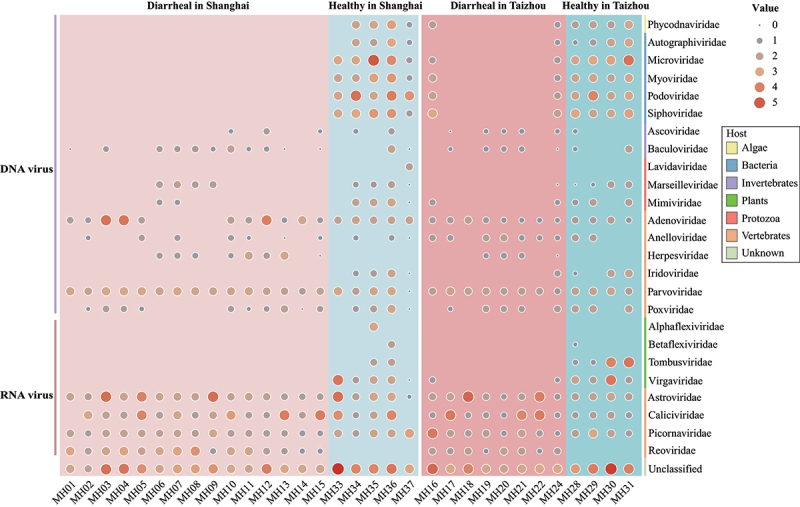
The composition of the gut viral communities at the family level. Bubble plot showing the relative abundance of each viral family of the individual library on a log10 scale. The viral genome composition was represented by different colored rectangles with taxon names indicated on the right. The groups and types of viral families are labeled and the host of each family is shown with corresponding colors (see color legend).

To reveal the different components of the gut viral communities, viral reads were identified at the family level and species level. At the family level, viral reads were classified into 25 viral families, consisting of 17 DNA viral families and 8 RNA viral families (Figure 2). The families *Adenoviridae* (66,662 reads, 9.03%), *Astroviridae* (204,063 reads, 27.6%), *Caliciviridae* (95,931 reads, 13.00%), and *Picornaviridae* (22,635 reads, 3.07%) were observed with relatively high abundance in all four groups, the natural hosts of which are vertebrates. Notably, the family *Reoviridae* (8,732 reads, 1.18%), which was then identified as rotavirus, was detected in the fecal samples from two diarrheal groups. At the same time, there were no reads detected from the two healthy groups. Phage *Autographiviridae* (1,734 reads, 0.23%), *Microviridae* (158,836 reads, 21.52%), *Myoviridae* (6,084 reads, 0.82%), *Podoviridae* (62,224 reads, 8.34%), and *Siphoviridae* (13,101 reads, 1.78%) accounted for high proportion, especially in the two healthy groups. The plant virus *Tombusviridae* (31,969 reads, 4.33%), and *Virgaviridae* (44,178 reads, 5.99%) were mainly detected in two healthy groups, indicating that a significant portion of intestinal RNA virus primarily derives from the diet. Surprisingly, *Microviridae*, *Myoviridae*, *Podoviridae*, and *Siphoviridae* were abundant in libraries MH16 and MH24 from group Diarrheal in Taizhou. The hierarchical clustering of 32 libraries based on the viral distribution at the family level showed similar patterns (Supplementary Fig. S1).

**Figure 3.**
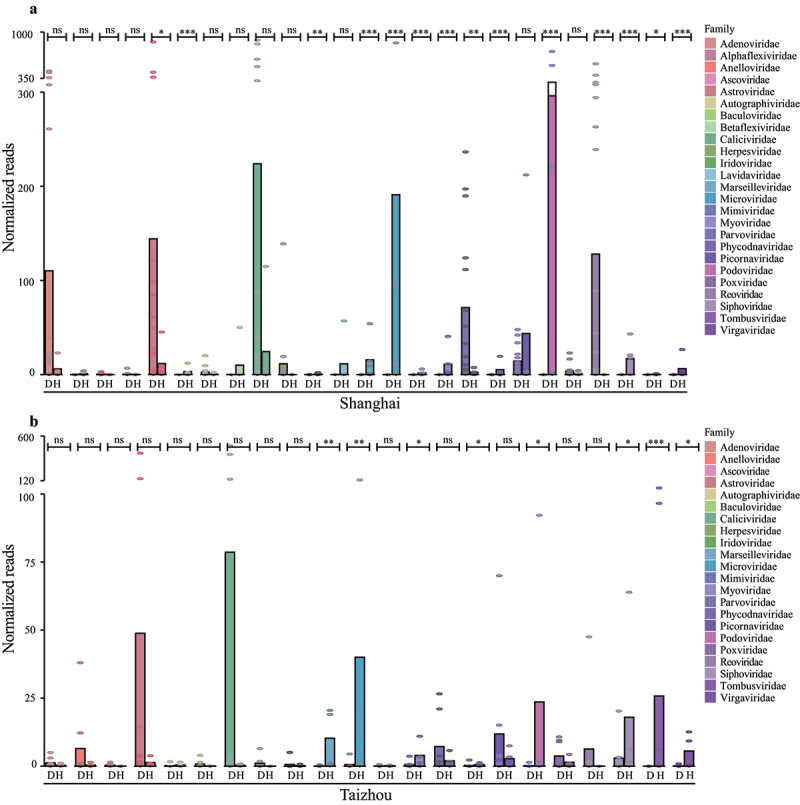
The comparison of the gut viral communities at the family level. Bar plots showing the normalized viral reads of families in each group by Megan. The viral families are shown with corresponding colors in the color legend. D stands for diarrheal group and H stands for healthy group. Each point represents one library and the difference between the two groups was measured by the Wilcoxon Mann Whitney test (*P < .05, **P < .01, and ***P < .001. ns indicates a nonsignificant difference).

Viral reads were normalized and compared to investigate the difference at the family level between diarrheal groups and healthy controls. In Shanghai, 14 families showed significant differences in abundance between the diarrheal group and the healthy group. In the diarrheal group, viral reads in the *Astroviridae*, *Parvoviridae*, and *Reoviridae* were significantly higher than healthy controls. Phages *Microviridae*, *Myoviridae*, *Podoviridae*, and *Siphoviridae*, and plant viruses *Tombusviridae* and *Virgaviridae* showed a higher abundance in the healthy group (Figure 3a). Notably, the families *Myoviridae*, *Podoviridae*, and *Siphoviridae* are members of the order *Caudovirales*. In Taizhou, eight families showed significant differences in abundance between the two groups. Phages and plant viruses showed significant differences in the healthy group (Figure 3b).

**Figure 4.**
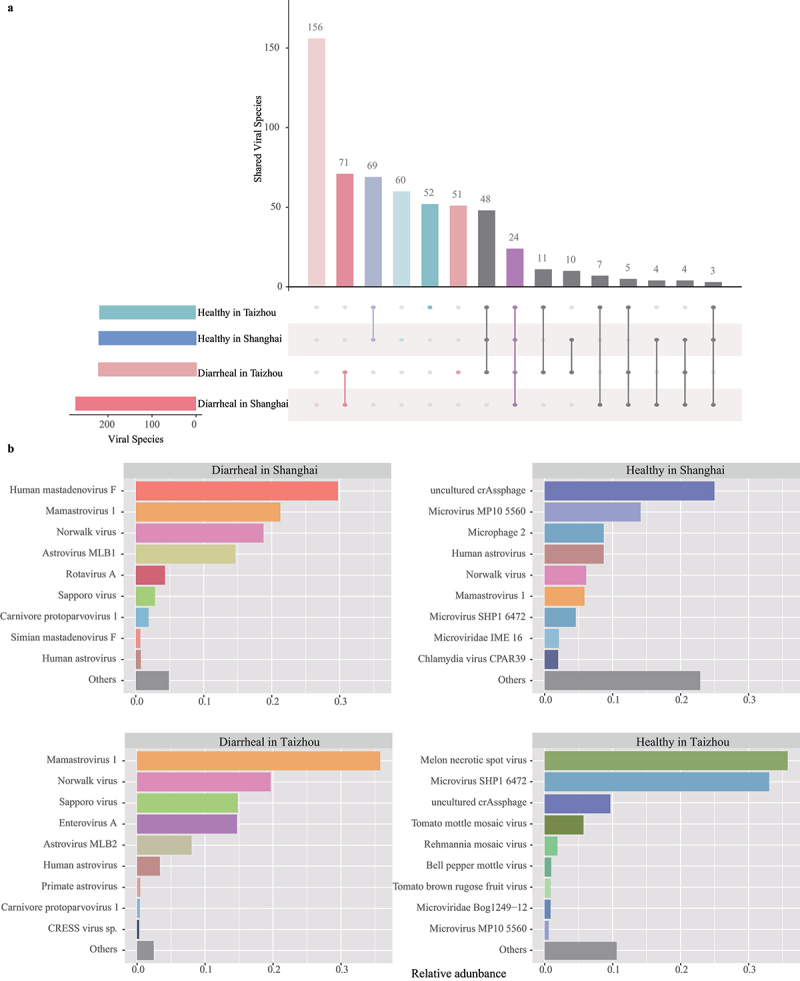
The composition of the gut viral communities at the species level. (a) UpSet plot showing the numbers of shared species in four groups. Filled dots with connected vertical lines represent the intersections, and unfilled dots in light gray represent sets that do not belong to the intersections. The vertical bars represent the number of viral species within the intersections, and the horizontal bars represent the total number of viral species in each group. (b) Bar plots of the top nine most abundant viral species in each group. The shared viral species among each group are indicated with the same color. The horizontal axis indicates the relative abundance of reads assigned to each viral species.

At the species level, viral reads were identified into 575 viral species in all four groups. The horizontal bar plot showed the number of viral species in four groups, in which the virome in group Diarrheal in Shanghai had the most diverse virus community. There were 35 viral species shared between two groups in Shanghai and 88 viral species shared between two groups in Taizhou, indicating the gut virome of two groups in Taizhou had a closer similarity (Figure 4a). In Shanghai, Human mastadenovirus F (29.73%) was the most prevalent virus in the diarrheal group, while uncultured crAssphage (24.99%) was the most dominant virus in the healthy group. In Taizhou, Mamastrovirus 1, the highest proportion of virus, accounted for 35.78% in the diarrheal group. Melon necrotic spot virus, a plant virus, accounted for 35.76% in group Healthy in Taizhou (Figure 4b). Surprisingly, in group Diarrheal in Shanghai, Rotavirus A was the fifth highest relative abundance in the gut virome. At the same time, Rotavirus A had a low relative abundance in group Diarrheal in Taizhou. The composition of the gut viral community in two diarrheal groups showed regional differences, which might be caused by different levels of rotavirus replication between patients in Shanghai and Taizhou. In two diarrheal groups, the gut virome mainly consisted of eukaryotic viruses. On the contrary, phages and plant viruses were major constituents of the gut viral communities in healthy groups.

**Figure 5.**
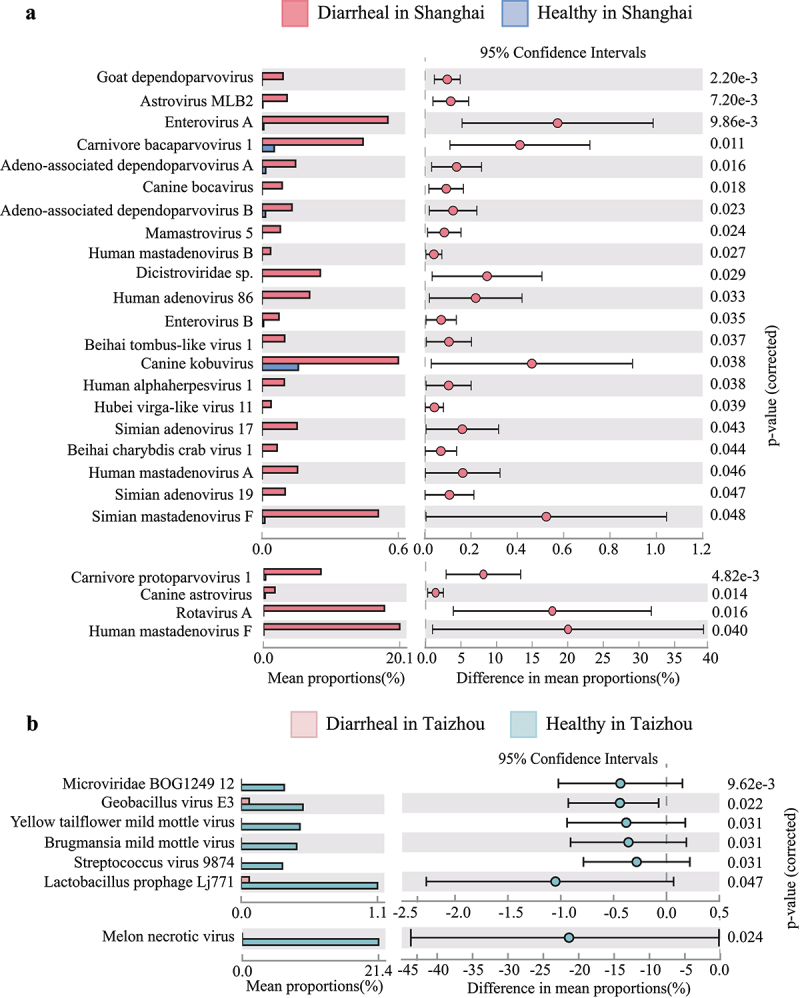
STAMP analyses of the gut virome at the species level. (a) Analysis of differences of viral communities in relative abundance between Diarrheal in Shanghai group and healthy in Shanghai group using STAMP. Legends are shown on the right. (b) Analysis of differences of viral communities in relative abundance between Diarrheal in Taizhou group and healthy in Taizhou group using STAMP. Legends are shown on the right.

Statistical Analysis of Metagenomic Profiles (STAMP) provides statistical hypothesis tests and exploratory plots.^20^ In this study, STAMP analyses showed differences in relative abundance in the gut virome at the species level between diarrheal and healthy groups. According to the results presented by STAMP, there were 25 differentiating species in two groups in Shanghai, most of which belonged to *Adenoviridae* and *Astroviridae* (Figure 5a). The relative abundances of seven viral species of phages and plant viruses were significantly different in the two groups in Taizhou (Figure 5b).

### Identification and analysis of enteric viruses

In this study, two trains of Rotavirus A were identified, named Human Rotavirus A fe01 and fe08. To investigate the genetic relationship of the two rotaviruses, phylogenetic analyses were performed based on the nucleotide sequences of the VP7 and VP4. The phylogenetic analyses identified Human Rotavirus A fe01 into G3P[8] genotype and Human Rotavirus A fe08 into G9P[8] genotype, which are two main predominant genotypes in China (Supplementary Fig. S2). The families *Adenoviridae*, *Astroviridae*, *Caliciviridae*, and *Picornaviridae* were identified with high abundance. Phylogenetic analyses were performed based on the nucleotide sequences of specific genes. The results showed that viruses identified from these four families were closely clustered with the virus strains that can infect humans and cause intestinal symptoms, respectively (Supplementary Fig. S3).

To determine the prevalence rate of rotaviruses, adenoviruses, astroviruses, noroviruses, and coxsackieviruses included in this study, all fecal samples were analyzed using the nPCR method. The prevalence rate of Rotavirus A had a significant difference between the diarrheal group and the healthy group in two places. Surprisingly, a small number of healthy children tested positive for RVA by the nested PCR method. Compared with the immune method, the nested PCR has higher sensitivity and a lower limit of detection, as a result of which, it was reasonable to be positive in the immunologically negative samples by nested PCR detection. In the group Diarrheal in Shanghai, the prevalence rates of Human Astrovirus MH18con1 (30.36%), Human Norovirus MH07con1 (15.79%), and Human Norovirus MH10con1 (19.74%) were significantly different from the group Healthy in Shanghai. In the group Diarrheal in Taizhou, Human Norovirus MH05con1 had a prevalence rate of 25%, which was significantly higher than the group Healthy in Taizhou. Surprisingly, in the group Healthy in Taizhou, the prevalence rate of Human Norovirus MH10con1 was 15.79% (Table 1).

**Table 1.** Comparison of molecular epidemiology of enteric viruses in different samples.

Virus	Shanghai				Taizhou			
	Diarrheal(%)	Healthy(%)	χ^2^ value	P value	Diarrheal(%)	Healthy(%)	χ^2^ value	P value
Rotavirus A	74(97.37)	2(7.41)	83.358	<.001	40(100.00)	4(21.05)	42.344	<.001
Adenovirus F	11(14.47)	1(3.70)	2.245	.250	4(10.00)	2(10.53)	–	1.000
Astrovirus 1	23(30.26)	3(11.11)	3.997	.046	7(17.50)	3(15.79)	–	1.000
Astrovirus 5	4(5.26)	1(3.70)	–	1.000	7(17.50)	0(0.00)	3.773	.131
Astrovirus MLB1	19(25.00)	3(11.11)	2.288	.130	8(20.00)	1(5.26)	2.164	.279
Norovirus GII.2	15(19.74)	14(51.85)	10.158	.010	0(0.00)	3(15.79)	–	.030
Norovirus GII.3	9(11.84)	2(7.41)	–	.781	10(25.00)	0(0.00)	5.719	.043
Norovirus GII.4	12(15.79)	12(44.44)	9.153	.002	5(12.50)	6(31.58)	3.091	.161
Coxsackievirus B5–1	17(22.37)	13(48.15)	6.414	.011	5(12.50)	2(10.53)	–	1.000
Coxsackievirus B5–2	8(10.53)	2(7.41)	–	.927	9(22.50)	3(15.79)	0.358	.801
Coxsackievirus B2	15(19.74)	6(22.22)	0.076	.783	11(27.50)	7(36.84)	0.530	.466
Coxsackievirus A4	12(15.79)	1(3.70)	–	.198	6(15.00)	0(0.00)	1.743	.187

The prevalence rate of each virus in each group was calculated and measured by the Chi-square test or Fisher’s exact probability text. - indicates the value calculated by Fisher’s exact probability method, without the chi-square value. *P* < .05 was considered statistically significant.

Phylogenetic analyses and epidemiological data suggested that enteric viruses were highly diverse in the human gut virome. The prevalence of these enteric viruses was much different in diarrheal children than in healthy children, despite previous results showing that the alpha diversity of the gut viral community in diarrheal groups was not different from controls.

### Identification and analysis of the gut phages

Phages are prevalent and diverse in all ecosystems. *Microviridae* are one of the most commonly retrieved viruses of single-stranded DNA phages, and *Caudovirales* are the most abundant and ubiquitous viruses with double-stranded genomes. Ten viral sequences of *Microviridae* and three viral sequences of *Caudovirales* were identified. Phylogenetic analyses were performed based on amino acid sequences of the virus hallmark genes of four families.^21^ The results showed the high diversity of *Microviridae* and *Caudovirales*, indicating that phages may play an essential role in the human gut ecosystem (Supplementary Fig. S4).

## Discussion

In this study, the diarrheal gut virome and the healthy gut virome were identified and compared. The alpha diversity of the diarrheal virome was not significantly different from that of the healthy virome. Meanwhile, the beta diversity significantly differed between diarrheal and healthy children. The diarrheal virome was mainly dominated by the family *Adenoviridae*, *Astroviridae*, *Caliciviridae*, and *Picornaviridae*, in addition to which, the healthy virome also contained phages including *Microviridae* and *Caudovirales*. Analyses of enteric viruses revealed the high diversity and differences in the distribution, and the little abundance of *Microviridae* and *Caudovirales* in diarrheal groups was identified. The study showed that diarrheal children had significant differences in the gut viral community composition, but the richness of viral species was consistent with healthy children.

The human gut microbiome is characterized by high diversity, gene richness, and stability. When it comes to perturbations such as infections and changes in the diet, a healthy gut microbiome is resilient to disturbance and able to recover the original composition whereas a non-resilient microbiome may shift to a maladaptive status of dysbiosis.^22^ The previous study showed that the *Coxsackievirus*-infected mouse gut virome changed vastly and had significant alterations in the alpha diversity and beta diversity during the progression of virus infection.^23^ Lu et al. identified a significantly lower alpha diversity of the gut virome in diarrheal dairy calves.^24^ The study by the team of Becerra revealed the dissimilarity of the gut virome of diarrheal children.^25^ The human virome has a more complex gut viral community than experimental mice and dairy calves and a better capacity of resisting disturbance. The difference in the gut virome in diarrheal samples could be caused by frequent washouts and watery stool, in which the loss of intestinal contents changed the internal environment of the intestine.^26^ Commensal with bacteria, phages were wiped out with the decrease of the host.

The virome in the human intestine consists of bacteriophages and eukaryotic viruses, in which phages are more abundant. In this study, DNA phages *Caudovirales* and *Microviridae* were dominated in the bacteriophage component with no RNA phages detected. The composition of the bacteriophages was consistent with studies by the team of Blanca Taboada^27^ and team of Yan.^28^ Tao Zhang found that more than 95% of RNA viruses in the healthy human intestine were likely plant-infecting viruses and might be ingested together with food.^29^ Li Hong’s study showed that a small proportion of *Levivirus*, a member of RNA phages, was identified in the gut virome in healthy children in Zhenjiang, Jiangsu.^30^ The presence of RNA phages was different from this study, which was caused by different living environments. However, according to available studies, gut phages are the most diversified and unrecognized component of the human microbial ecosystem. A great majority of novel sequences named viral dark matter have no or little homology to public databases. There might be RNA phages identified in the human gut with the development of viral databases.

The order *Caudovirales* predominates in early infancy and decreases while the family *Microviridae* increase in childhood, during which time both phages remain dominant, and the abundant content may mean that they take on more functions.^31^ Phages function by directly affecting the host immune or indirectly altering the gut bacterial composition and influencing the gut bacteriome activity.^32^ Recently, the study on filtered fecal transplants has raised, in which the viral portion of the fecal sample is isolated and implanted in another host. Transplantation of fecal viromes from healthy mice could make obese mice with type 2 diabetes weight loss and normalized blood glucose parameters.^33^ Sterile filtrates from healthy human stools could improve symptoms in patients with Clostridium difficile.^34^ The specific change of phages in children with diarrhea provides a reference for the clinical treatment of fecal virome transplantation for diarrhea in the future, which may improve intestinal function.

Besides Rotavirus A, enteric viruses of the families *Adenoviridae* (adenoviruses), *Astroviridae* (astroviruses), *Caliciviridae* (noroviruses), and *Picornaviridae* (coxsackieviruses) were identified in both diarrheal groups and healthy groups, while most of them had no difference in the prevalence rate among diarrheal and healthy samples. In a study of the gut RNA virome of healthy infants, rotaviruses, echoviruses, and coxsackieviruses were found.^35^ A high prevalence rate of caliciviruses was detected in asymptomatic children in a study of determining the fecal virome of infants in Mexico.^36^ These studies indicate that many enteric infections happen during early childhood without severe clinical symptoms. Diverse enteric viruses were detected in combination with one another in both diarrheal children and healthy children. The high prevalence of enteric viruses provides a reference for the prevention of enteric virus infections.

In conclusion, this study first compared the gut virome between diarrheal children and healthy children. The study revealed a significant difference in the beta diversity of the viral community and identified the low abundance of *Caudovirales* and *Microviridae* in the diarrheal groups. This study deepened the knowledge of the influence of diarrhea on the human gut virome and revealed an extensive prevalence of diverse enteric viruses in children. The study introduced a general overview of the viral community inhabiting the children intestine and provided a reference for efficient treatments and prevention of virus-infectious illnesses including diarrhea in children.

## Materials and methods

### Sample collection and preparation

In 2018, 40 fecal samples from children with diarrhea and 19 fecal samples from healthy controls were collected at The Affiliated Taizhou People’s Hospital of Nanjing Medical University in Jiangsu. In 2018, 76 fecal samples from children with diarrhea and 27 fecal samples from healthy controls were collected at Children’s Hospital of Shanghai. The inclusion criteria of diarrheal children were under 5 y of age, with three or more episodes of diarrhea in the preceding 24 h and positive on the stool for Group A Rotavirus (RVA) by colloidal gold immunochromatography. The inclusion criteria of healthy children were under 5 y of age, no diarrheal symptoms, and negative on the stool for Group A Rotavirus (RVA) by colloidal gold immunochromatography. Children were excluded if they had a known organic disorder, had any gastrointestinal surgery, or had active bacterial infections (including *Escherichia*, *Salmonella*, *Shigella*, *Vibrio cholera* et al.) or used immunosuppressor or antibiotics within 2 months.^28^ All samples tested negative for bacteria (including *Escherichia*, *Salmonella*, *Shigella*, *Vibrio cholera et al*.), mycoplasma, and Chlamydia.

Each sample was collected in a 1.5 mL sterile centrifuge tube and added 1 mL Dulbecco’s phosphate-cushioned saline (DPBS). All samples were frozen at −80°C for 30 min, thawed at room temperature for 30 min for three cycles, and then vortexed for 15 min tremendously.^37–39^ The supernatants were collected after centrifugation (5 min, 15000 g, 4°C). Fecal samples were combined into 32 pools according to the sampling source and stored at −80°C.

### Viral metagenomic analysis

Each supernatant pool (500 μL) was filtered through a 0.45 μm filter (Millipore) to remove eukaryotic and bacterial cell-sized particles.^40,41^ The filtrates were then treated at 37°C for 90 min with DNase (Ambion) and RNase (Fermentas) to digest unprotected nucleic acids.^37,42,43^ The remaining total nucleic acids were extracted using a QIAamp Viral RNA Mini Kit (QIAGEN) according to the protocol. The nucleic acid exactions were treated with SuperScript III Reverse Transcriptase (Thermo) with six-base random primers to reverse RNA into cDNA, followed by a single round of DNA synthesis using Klenow fragment polymerase (New England BioLabs) to synthesize the second strand of single-stranded DNA. The resulting double-stranded DNA products were used to construct 32 libraries, using a Nextera XT DNA Sample Preparation Kit (Illumina) and then sequenced using an Illumina NovaSeq platform with 250 base pair-ends with dual barcoding for each individual library.^44,45^

### Bioinformatics analysis

Paired-end reads of 250 bp were generated by NovaSeq sequencing and then debarcoded for each DNA library by vendor software from Illumina. An in-house analysis pipeline running on a 32-node Linux cluster was utilized to process data. Reads were considered duplicates if bases 5–55 were identical, and then only one random copy of duplicates was kept and clonal reads were removed. Low-sequencing-quality tails were trimmed with a quality of score 30 as the threshold using Phred. Adaptors were trimmed using the default parameters of VecScreen in the National Center for Biotechnology Information (NCBI) (https://www.ncbi.nlm.nih.gov/tools/vecscreen/). The cleaned reads were assembled *de novo* within each barcode using Geneious Prime v2019.2.3.^46^ Contigs and singlets were matched against an in-house viral proteome database using BLASTx with an E-value of <10^−5^.^47,48^

### Phylogenetic analysis

Phylogenetic analyses were performed based on the nucleotide and amino acid sequences of viruses identified in this study and their best BLASTn matches in NCBI GenBank database as well as representative sequences from the corresponding family. Putative viral open reading frames (ORFs) were predicted by Geneious Prime v2019.2.3. Sequence alignment was performed using MUSCLE in MEGA v10.1.8 with default settings^49^ and phylogenetic trees were then constructed using MrBayes v3.2.7 by mixed models and Markov chain Monte Carlo (MCMC) methods. The runs were terminated until the average standard deviation of split frequencies was less than 0.01.^50^ Bayesian inference trees were visualized by Figtree v1.4.4 and Adobe Illustrator 2020 v24.0.1.

### Nested PCR

To detect the prevalence of enteric viruses found in this study, specific nested PCR primers were designed based on the results of phylogenetic analyses of these viruses. Strains clustered in one clade were arranged to the same primer for sharing sequences after sequence alignment. The PCR conditions were as follows: 95°C for 5 min, 30 cycles of 95°C for 30 s, 50°C (for the first round) or 55°C (for the second round) for 30 s, and 72°C for 15 s, a final extension at 72°C for 10 min, and the premixed enzyme rTaq (Takara) was added to the reaction system. Primers designed for this study are shown in Supplementary Table S2.

### Statistical analysis

All statistical analyses were performed by Megan v6.19.9, R v4.1.1, STAMP v2.1.3, and SPSS v26.0. The composition analyses were normalized using Megan.^51^ The viral community analyses were compared by R v4.1.1 package pheatmap, vegan, UpSetR, ggpubr, ggcharts, and ggplot2. The alpha diversity and beta diversity of the gut virome were compared by the Wilcoxon test. The comparison in diarrheal and healthy groups was measured by the Wilcoxon Mann–Whitney test. STAMP analysis was performed to analyze the difference in viral communities between diarrheal samples and healthy controls by White’s non-parametric t-test.^20^ Statistical analyses for the prevalence of enteric viruses were performed by SPSS v26.0 using the Chi-square test or Fisher’s exact probability test. *P* < .05 was considered statistically significant.

### Quality control

To prevent cross-contamination and nucleic acid degradation, standard precautions were performed during the whole process and all the materials used were DNase and RNase-free. Nucleic acid samples were dissolved in Water-DEPC Treated Water (Sangon Biotech) with RNase inhibitors added. Sterile ddH2O (Sangon Biotech) was prepared and further processed under the same conditions as a blank control. All experiments were performed in a Class II biological safety cabinet (Telstar).

## Supplementary Material

Supplemental Material

## Data Availability

The raw sequence reads generated by Illumina in the study are available at the NCBI Sequence Read Archive database under the Biosample accession SAMN33574712 (https://www.ncbi.nlm.nih.gov/biosample/SAMN33574712/), and BioProject accession PRJNA940581 (https://www.ncbi.nlm.nih.gov/bioproject/PRJNA940581) and the accession numbers are listed in Supplementary Table S1. All viral sequences identified in this study were deposited in the GenBank database, and the accession numbers were listed in Supplementary Table S3.
