# Supplementary material for: Viral metagenomics of the gut virome of diarrheal children with Rotavirus A infection
Source: Gut Microbes. 2023 Jul 13;15(1):2234653. doi: 10.1080/19490976.2023.2234653 (PMC10351451; doi:10.1080/19490976.2023.2234653)
Supplement: Supplemental Material [file KGMI_A_2234653_SM9927.zip › Supplemental material/Supplementary Table S1 clean.docx]

**Table S1.** Detailed information of 32 libraries constructed in this study.

| **Library** | **Group** | **Sampling source** | **Sampling date** | **Sample type** | **Sample number No.** | **age** | **Sample numbers** | **raw reads** | **viral reads** | **SRA Accession No.** |
| --- | --- | --- | --- | --- | --- | --- | --- | --- | --- | --- |
| MH01 | Diarrheal | Shanghai | 2018 | feces | 1-5 | children(<5 year) | 5 | 117440 | 1612 | SRR23692278 |
| MH02 | Diarrheal | Shanghai | 2018 | feces | 6-10 | children(<5 year) | 5 | 25172 | 900 | SRR23692292 |
| MH03 | Diarrheal | Shanghai | 2018 | feces | 11-15 | children(<5 year) | 5 | 402430 | 72436 | SRR23692766 |
| MH04 | Diarrheal | Shanghai | 2018 | feces | 16-20 | children(<5 year) | 5 | 335780 | 25157 | SRR23692928 |
| MH05 | Diarrheal | Shanghai | 2018 | feces | 21-25 | children(<5 year) | 5 | 255604 | 20238 | SRR23693716 |
| MH06 | Diarrheal | Shanghai | 2018 | feces | 26-30 | children(<5 year) | 5 | 244498 | 1210 | SRR23693715 |
| MH07 | Diarrheal | Shanghai | 2018 | feces | 31-35 | children(<5 year) | 5 | 498522 | 3605 | SRR23693702 |
| MH08 | Diarrheal | Shanghai | 2018 | feces | 36-40 | children(<5 year) | 5 | 315512 | 4275 | SRR23693932 |
| MH09 | Diarrheal | Shanghai | 2018 | feces | 41-45 | children(<5 year) | 5 | 235018 | 40122 | SRR23694014 |
| MH10 | Diarrheal | Shanghai | 2018 | feces | 46-50 | children(<5 year) | 5 | 254354 | 3045 | SRR23699152 |
| MH11 | Diarrheal | Shanghai | 2018 | feces | 51-55 | children(<5 year) | 5 | 190348 | 1287 | SRR23699153 |
| MH12 | Diarrheal | Shanghai | 2018 | feces | 56-60 | children(<5 year) | 5 | 210416 | 12687 | SRR23699151 |
| MH13 | Diarrheal | Shanghai | 2018 | feces | 61-65 | children(<5 year) | 5 | 109260 | 15032 | SRR23699158 |
| MH14 | Diarrheal | Shanghai | 2018 | feces | 66-70 | children(<5 year) | 5 | 11140 | 490 | SRR23699305 |
| MH15 | Diarrheal | Shanghai | 2018 | feces | 71-76 | children(<5 year) | 6 | 86800 | 15943 | SRR23699539 |
| MH16 | Diarrheal | Jiangsu, Taizhou | 2018 | feces | T1-T5 | children(<5 year) | 5 | 336796 | 19635 | SRR23699540 |
| MH17 | Diarrheal | Jiangsu, Taizhou | 2018 | feces | T6-T10 | children(<5 year) | 5 | 142372 | 18652 | SRR23699541 |
| MH18 | Diarrheal | Jiangsu, Taizhou | 2018 | feces | T11-T15 | children(<5 year) | 5 | 168762 | 60754 | SRR23699543 |
| MH19 | Diarrheal | Jiangsu, Taizhou | 2018 | feces | T16-T20 | children(<5 year) | 5 | 118552 | 749 | SRR23699544 |
| MH20 | Diarrheal | Jiangsu, Taizhou | 2018 | feces | T21-T25 | children(<5 year) | 5 | 75988 | 505 | SRR23699545 |
| MH21 | Diarrheal | Jiangsu, Taizhou | 2018 | feces | T26-T30 | children(<5 year) | 5 | 114648 | 8212 | SRR23699547 |
| MH22 | Diarrheal | Jiangsu, Taizhou | 2018 | feces | T31-T35 | children(<5 year) | 5 | 107624 | 30311 | SRR23699548 |
| MH24 | Diarrheal | Jiangsu, Taizhou | 2018 | feces | T41-T45 | children(<5 year) | 5 | 38204 | 287 | SRR23699549 |
| MH28 | Healthy | Jiangsu, Taizhou | 2018 | feces | T1H-T5H | children(<5 year) | 5 | 110876 | 2813 | SRR23699550 |
| MH29 | Healthy | Jiangsu, Taizhou | 2018 | feces | T6H-T10H | children(<5 year) | 5 | 533214 | 11920 | SRR23699580 |
| MH30 | Healthy | Jiangsu, Taizhou | 2018 | feces | T11H-T15H | children(<5 year) | 5 | 1284506 | 31903 | SRR23699615 |
| MH31 | Healthy | Jiangsu, Taizhou | 2018 | feces | T16H-T19H | children(<5 year) | 4 | 767786 | 57660 | SRR23699616 |
| MH33 | Healthy | Shanghai | 2018 | feces | 6H-10H | children(<5 year) | 5 | 2017958 | 65562 | SRR23699617 |
| MH34 | Healthy | Shanghai | 2018 | feces | 11H-15H | children(<5 year) | 5 | 497732 | 36360 | SRR23699628 |
| MH35 | Healthy | Shanghai | 2018 | feces | 16H-20H | children(<5 year) | 5 | 1687500 | 126919 | SRR23699646 |
| MH36 | Healthy | Shanghai | 2018 | feces | 21H-25H | children(<5 year) | 5 | 1618790 | 42111 | SRR23699647 |
| MH37 | Healthy | Shanghai | 2018 | feces | 26H-32H | children(<5 year) | 7 | 22690 | 5604 | SRR23699648 |
