# Supplementary material for: Viral metagenomics of the gut virome of diarrheal children with Rotavirus A infection
Source: Gut Microbes. 2023 Jul 13;15(1):2234653. doi: 10.1080/19490976.2023.2234653 (PMC10351451; doi:10.1080/19490976.2023.2234653)
Supplement: Supplemental Material [file KGMI_A_2234653_SM9927.zip › Supplemental material/Supplementary Table S2.docx]

**Table S2.** The primers of two rounds of nested PCR

| Virus | Primer name | Application | Primer sequence (5’ to 3’) |
| --- | --- | --- | --- |
| Rotavirus A | RV-VP6-WF | 1st round | ACAATTCGCCTGTCATTCCA |
|  | RV-VP6-WR | 1st round | ACATGCTTCTGATAGAGGCT |
|  | RV-VP6-NF | 2nd round | TGCAACAGTTGGACTTACGT |
|  | RV-VP6-NR | 2nd round | GCTCAGTCCAATTCATACCTGG |
| Adenovirus F | ADV-WF | 1st round | AGGTGTCCATCATGTTCGACT |
|  | ADV-WR | 1st round | ACCTGACGACTCATTGGCTG |
|  | ADV-NF | 2nd round | TGGCAATGACCGACTGCTTA |
|  | ADV-NR | 2nd round | CCAATGTTGTAGTGACTAAGCA |
| Astrovirus 1 | AST1-WF | 1st round | GAGGACGAAGACGACGAAGC |
|  | AST1-WR | 1st round | CGAGACAAGCAGGTCCATGT |
|  | AST1-NF | 2nd round | GAAGACGACGAAGCGGACAG |
|  | AST1-NR | 2nd round | CATTGTCATTCCTTGGTTTACG |
| Astrovirus 5 | AST5-WF | 1st round | ATCAACACTGCCGACGTCTT |
|  | AST5-WR | 1st round | AGTTGTGGTGACTGCAGACG |
|  | AST5-NF | 2nd round | TGTCTGCCGCTGAATTGGTT |
|  | AST5-NR | 2nd round | AGCTACGCGTACCATTCCTT |
| Astrovirus MLB1 | ASTM-WF | 1st round | CCAAGATCCATTGTCACACC |
|  | ASTM-WR | 1st round | AAGCCGGTCAACAAGAACCA |
|  | ASTM-NF | 2nd round | AGCTATCCAATGACACCAAG |
|  | ASTM-NR | 2nd round | ACCTTAGAAGTGTTCTGTTGGA |
| Norovirus GⅡ.2 | NOVⅡ.2-WF | 1st round | GCGCCTGCTCTTCTTCAGAT |
|  | NOVⅡ.2-WR | 1st round | GGTTGATGTATCTGACGAGG |
|  | NOVⅡ.2-NF | 2nd round | CCAGCCATTGATTGCCTACT |
|  | NOVⅡ.2-NR | 2nd round | GGTGTTGCTCGAGACTGTCA |
| Norovirus GⅡ.3 | NOVⅡ.3-WF | 1st round | CCTGGTGAAGTGCTCCTCAA |
|  | NOVⅡ.3-WR | 1st round | AGCTGCCTGACATCCACAAT |
|  | NOVⅡ.3-NF | 2nd round | TGTATAATGGTTATGCAGGTGG |
|  | NOVⅡ.3-NR | 2nd round | CTGTGCTGCACTTAGATTGT |
| Norovirus GⅡ.4 | NOVⅡ.4-WF | 1st round | TTCTTGCCGAGTTCTCACGA |
|  | NOVⅡ.4-WR | 1st round | CCATCAGTCGTGCACCTACC |
|  | NOVⅡ.4-NF | 2nd round | TTGCCGAGTTCTCACGAGAC |
|  | NOVⅡ.4-NR | 2nd round | TGGTCATCTCCTCAACAGTT |
| Coxsackievirus B5-1 | COXB5-1-WF | 1st round | AAGGCCAGGATTCACCAGTG |
|  | COXB5-1-WR | 1st round | GCTGCCATCATTCACGTGTC |
|  | COXB5-1-NF | 2nd round | CTACAGCTGGCAGACGTCCA |
|  | COXB5-1-NR | 2nd round | ACCATATGTTCCTTGCTTGT |
| Coxsackievirus B5-2 | COXB5-2-WF | 1st round | AAGCCGTTGATCACTACGCT |
|  | COXB5-2-WR | 1st round | CACATAGGTCACCATCGGCA |
|  | COXB5-2-NF | 2nd round | TGGAAGCATTGGACTTGACCA |
|  | COXB5-2-NR | 2nd round | CCATACACTCCTTCAGCTTGGT |
| Coxsackievirus B2 | COXB2-WF | 1st round | AATCATGACGCCAGCAGACA |
|  | COXB2-WR | 1st round | GACACAATGACCGCACATGG |
|  | COXB2-NF | 2nd round | GGTCACATGGACTAATGTCACT |
|  | COXB2-NR | 2nd round | TGTCCACCTAATAGATTCGTGA |
| Coxsackievirus A4 | COXB2-WF | 1st round | GCAGAGACTGGAGCCTCATC |
|  | COXB2-WR | 1st round | CACACTGTTGTTCGTGAGCC |
|  | COXB2-NF | 2nd round | GTGTTATGGAGGTGGACGAC |
|  | COXB2-NR | 2nd round | ACGAAGGTGAATTCTGCATC |
