# Supplementary material for: Viral metagenomics of the gut virome of diarrheal children with Rotavirus A infection
Source: Gut Microbes. 2023 Jul 13;15(1):2234653. doi: 10.1080/19490976.2023.2234653 (PMC10351451; doi:10.1080/19490976.2023.2234653)
Supplement: Supplemental Material [file KGMI_A_2234653_SM9927.zip › Supplemental material/Supplementary Table S3.docx]

Table S3. Detailed information of viral sequences identified in this study

| **Virus strain name** | **GenBank No.** | SRA Accession No. | **Length** | **Virus Reads** | **Virus hallmark genes** | **Completeness of genome** | **Classification** | **Blastx hits on known protein and viruses** | **Identity (%)** | **Accession numbers  of best match** |
| --- | --- | --- | --- | --- | --- | --- | --- | --- | --- | --- |
| Human Rotavirus A fe01 VP4 (VP4) gene, partial cds | OQ633112 | SRR23692278 | 2223 | 2911 | VP4 | partial | Reoviridae | [VP4 protein [Human rotavirus A]](https://blast.ncbi.nlm.nih.gov/Blast.cgi#alnHdr_BBM12425) | 99.73 | [BBM12425.1](https://www.ncbi.nlm.nih.gov/protein/BBM12425.1?report=genbank&log$=prottop&blast_rank=1&RID=Z3SDFJ06016) |
| Human Rotavirus A fe01 VP7 (VP7) gene, partial cds | OQ633113 | SRR23692278 | 735 | 32 | VP7 | partial | Reoviridae | [VP7 [Rotavirus A]](https://blast.ncbi.nlm.nih.gov/Blast.cgi#alnHdr_UTQ10391) | 100.00 | [UTQ10391.1](https://www.ncbi.nlm.nih.gov/protein/UTQ10391.1?report=genbank&log$=prottop&blast_rank=1&RID=Z3T6N8CH016) |
| Human Rotavirus A fe08 VP4 (VP4) gene, partial cds | OQ633114 | SRR23693932 | 2381 | 261 | VP4 | partial | Reoviridae | [outer capsid protein VP4 [Human rotavirus A]](https://blast.ncbi.nlm.nih.gov/Blast.cgi#alnHdr_BCK33991) | 99.87 | [BCK33991.1](https://www.ncbi.nlm.nih.gov/protein/BCK33991.1?report=genbank&log$=prottop&blast_rank=1&RID=Z3SENBUY013) |
| Human Rotavirus A fe08 VP7 (VP7) gene, partial cds | OQ633115 | SRR23693932 | 1002 | 1285 | VP7 | partial | Reoviridae | [major outer capsid protein VP7 [Human rotavirus A]](https://blast.ncbi.nlm.nih.gov/Blast.cgi#alnHdr_AHC72920) | 99.69 | [AHC72920.1](https://www.ncbi.nlm.nih.gov/protein/AHC72920.1?report=genbank&log$=prottop&blast_rank=1&RID=Z3T7611B016) |
| Human Adenovirus MH03con1 hexon protein gene, complete cds | OQ633090 | SRR23692766 | 2778 | 20923 | Hexon protein | complete | Adenoviridae | hexon [Human adenovirus 41] | 100 | [AQR58944.1](https://www.ncbi.nlm.nih.gov/protein/AQR58944.1?report=genbank&log$=prottop&blast_rank=1&RID=Z3TNHZ3H01R) |
| Human Adenovirus MH04con1 hexon protein gene, complete cds | OQ633091 | SRR23692928 | 2778 | 20886 | Hexon protein | complete | Adenoviridae | hexon [Human adenovirus 42] | 100 | [ADN06455.1](https://www.ncbi.nlm.nih.gov/protein/ADN06455.1?report=genbank&log$=prottop&blast_rank=1&RID=Z3TP2XCZ01R) |
| Human Astrovirus MH03con2 | OQ633094 | SRR23692766 | 6736 | 38602 | capsid protein | complete | Astroviridae | [putative serine protease [Astrovirus MLB1]](https://blast.ncbi.nlm.nih.gov/Blast.cgi#alnHdr_USJ75243) | 99.87 | [USJ75243.1](https://www.ncbi.nlm.nih.gov/protein/USJ75243.1?report=genbank&log$=prottop&blast_rank=1&RID=Z44CMPMA01R) |
| Human Astrovirus MH09con1 | OQ633093 | SRR23694014 | 6835 | 88643 | capsid protein | complete | Astroviridae | [erine protease [Human astrovirus]](https://blast.ncbi.nlm.nih.gov/Blast.cgi#alnHdr_QBL55997) | 99.57 | [QBL55997.1](https://www.ncbi.nlm.nih.gov/protein/QBL55997.1?report=genbank&log$=prottop&blast_rank=1&RID=Z44DKDYC01R) |
| Human Astrovirus MH18con1 | OQ633092 | SRR23699543 | 6826 | 122612 | capsid protein | complete | Astroviridae | [nonstructural protein [Human astrovirus]](https://blast.ncbi.nlm.nih.gov/Blast.cgi#alnHdr_QWY12971) | 99.13 | [QWY12971.1](https://www.ncbi.nlm.nih.gov/protein/QWY12971.1?report=genbank&log$=prottop&blast_rank=1&RID=Z44DXBJ401R) |
| Human Norovirus MH07con1 | OQ633111 | SRR23693702 | 3309 | 29893 | capsid protein | partial | Caliciviridae | [capsid protein [Norovirus GII.4]](https://blast.ncbi.nlm.nih.gov/Blast.cgi#alnHdr_ATI15323) | 99.81 | [ATI15323.1](https://www.ncbi.nlm.nih.gov/protein/ATI15323.1?report=genbank&log$=prottop&blast_rank=1&RID=Z44KZ8SM01R) |
| Human Norovirus MH10con1 | OQ633109 | SRR23699152 | 7429 | 998 | capsid protein | partial | Caliciviridae | [nonstructural polyprotein [Norovirus GII]](https://blast.ncbi.nlm.nih.gov/Blast.cgi#alnHdr_AUY62140) | 99.76 | [AUY62140.1](https://www.ncbi.nlm.nih.gov/protein/AUY62140.1?report=genbank&log$=prottop&blast_rank=1&RID=Z44MH39G01R) |
| Human Norovirus MH17con4 | OQ633110 | SRR23699541 | 7228 | 584 | capsid protein | partial | Caliciviridae | [nonstructural polyprotein [Norovirus GII]](https://blast.ncbi.nlm.nih.gov/Blast.cgi#alnHdr_UJP38444) | 100 | [UJP38444.1](https://www.ncbi.nlm.nih.gov/protein/UJP38444.1?report=genbank&log$=prottop&blast_rank=1&RID=Z44MZYVE01R) |
| Human Coxsackievirus MH16con1 | OQ633098 | SRR23699540 | 7346 | 115283 | RNA dependent RNA polymerase | complete | Picornaviridae | [polyprotein [Coxsackievirus A4]](https://blast.ncbi.nlm.nih.gov/Blast.cgi#alnHdr_QKE30983) | 99.45 | [QKE30983.1](https://www.ncbi.nlm.nih.gov/protein/QKE30983.1?report=genbank&log$=prottop&blast_rank=1&RID=Z44SDA1Y01R) |
| Human Coxsackievirus MH28con1 | OQ633095 | SRR23699550 | 6452 | 1381 | RNA dependent RNA polymerase | partial | Picornaviridae | [polyprotein [Coxsackievirus B5]](https://blast.ncbi.nlm.nih.gov/Blast.cgi#alnHdr_UUG59705) | 99.85 | [UUG59705.1](https://www.ncbi.nlm.nih.gov/protein/UUG59705.1?report=genbank&log$=prottop&blast_rank=1&RID=Z44TFRDX01R) |
| Human Coxsackievirus MH29con1 | OQ633096 | SRR23699580 | 6851 | 15137 | RNA dependent RNA polymerase | partial | Picornaviridae | [polyprotein [Coxsackievirus B5]](https://blast.ncbi.nlm.nih.gov/Blast.cgi#alnHdr_ASY05915) | 99.31 | [ASY05915.1](https://www.ncbi.nlm.nih.gov/protein/ASY05915.1?report=genbank&log$=prottop&blast_rank=1&RID=Z44TXRPZ01R) |
| Human Coxsackievirus MH35con1 | OQ633097 | SRR23699646 | 6545 | 813 | RNA dependent RNA polymerase | partial | Picornaviridae | [polyprotein [Coxsackievirus B2]](https://blast.ncbi.nlm.nih.gov/Blast.cgi#alnHdr_AOW42548) | 98.71 | [AOW42548.1](https://www.ncbi.nlm.nih.gov/protein/AOW42548.1?report=genbank&log$=prottop&blast_rank=1&RID=Z44UERPE01R) |
| Microvirus strain 07-01 | OQ633099 | SRR23693702 | 5371 | 3176 | major capsid protein | complete | Micviridae | [TPA: major capsid protein [Microviridae sp.]](https://blast.ncbi.nlm.nih.gov/Blast.cgi#alnHdr_DAR35672) | 74.6 | [DAR35672.1](https://www.ncbi.nlm.nih.gov/protein/DAR35672.1?report=genbank&log$=prottop&blast_rank=1&RID=Z45ATV5U016) |
| Microvirus strain 19-01 | OQ633100 | SRR23699544 | 5310 | 1246 | major capsid protein | complete | Micviridae | [VP1 [Gokushovirus WZ-2015a]](https://blast.ncbi.nlm.nih.gov/Blast.cgi#alnHdr_ALS03631) | 95.34 | [ALS03631.1](https://www.ncbi.nlm.nih.gov/protein/ALS03631.1?report=genbank&log$=prottop&blast_rank=2&RID=Z45B5H8A016) |
| Microvirus strain 20-01 | OQ633101 | SRR23699545 | 2941 | 329 | major capsid protein | partial | Micviridae | [major capsid protein [Microviridae sp.]](https://blast.ncbi.nlm.nih.gov/Blast.cgi#alnHdr_AXL15119) | 87.48 | [AXL15119.1](https://www.ncbi.nlm.nih.gov/protein/AXL15119.1?report=genbank&log$=prottop&blast_rank=1&RID=Z45BHVYR013) |
| Microvirus strain 29-01 | OQ633102 | SRR23699580 | 6032 | 26044 | major capsid protein | complete | Micviridae | [TPA: Replication associated protein [Microviridae sp.]](https://blast.ncbi.nlm.nih.gov/Blast.cgi#alnHdr_DAX06273) | 93.37 | [DAX06273.1](https://www.ncbi.nlm.nih.gov/protein/DAX06273.1?report=genbank&log$=prottop&blast_rank=1&RID=Z45BVTP8016) |
| Microvirus strain 31-01 | OQ633103 | SRR23699616 | 6364 | 6364 | major capsid protein | complete | Micviridae | [TPA: Major capsid protein [Microviridae sp.]](https://blast.ncbi.nlm.nih.gov/Blast.cgi#alnHdr_DAT92652) | 93.11 | [DAT92652.1](https://www.ncbi.nlm.nih.gov/protein/DAT92652.1?report=genbank&log$=prottop&blast_rank=1&RID=Z45C6HVE016) |
| Microvirus strain 33-01 | OQ633104 | SRR23699617 | 5190 | 765 | major capsid protein | partial | Micviridae | [TPA: major capsid protein [Microviridae sp.]](https://blast.ncbi.nlm.nih.gov/Blast.cgi#alnHdr_DAM38707) | 87.94 | [DAM38707.1](https://www.ncbi.nlm.nih.gov/protein/DAM38707.1?report=genbank&log$=prottop&blast_rank=1&RID=Z45CP11F013) |
| Microvirus strain 34-01 | OQ633105 | SRR23699628 | 4887 | 351 | major capsid protein | partial | Micviridae | [TPA: Major capsid protein [Microviridae sp.]](https://blast.ncbi.nlm.nih.gov/Blast.cgi#alnHdr_DAW48344) | 85.74 | [DAW48344.1](https://www.ncbi.nlm.nih.gov/protein/DAW48344.1?report=genbank&log$=prottop&blast_rank=1&RID=Z45CZW7B013) |
| Microvirus strain 35-01 | OQ633106 | SRR23699646 | 5656 | 268163 | major capsid protein | complete | Micviridae | [TPA: Major capsid protein [Microviridae sp.]](https://blast.ncbi.nlm.nih.gov/Blast.cgi#alnHdr_DAM63148) | 84.5 | [DAM63148.1](https://www.ncbi.nlm.nih.gov/protein/DAM63148.1?report=genbank&log$=prottop&blast_rank=1&RID=Z45DDBPU013) |
| Microvirus strain 35-12 | OQ633107 | SRR23699646 | 5252 | 14985 | major capsid protein | partial | Micviridae | [TPA: major capsid protein [Microviridae sp. ctCuG14]](https://blast.ncbi.nlm.nih.gov/Blast.cgi#alnHdr_DAD85233) | 95.13 | [DAD85233.1](https://www.ncbi.nlm.nih.gov/protein/DAD85233.1?report=genbank&log$=prottop&blast_rank=1&RID=Z45DRHWZ016) |
| Microvirus strain 36-01 | OQ633108 | SRR23699647 | 6604 | 21892 | major capsid protein | complete | Micviridae | [TPA: Major capsid protein [Microviridae sp.]](https://blast.ncbi.nlm.nih.gov/Blast.cgi#alnHdr_DAX07558) | 85.87 | [DAX07558.1](https://www.ncbi.nlm.nih.gov/protein/DAX07558.1?report=genbank&log$=prottop&blast_rank=1&RID=Z45E40GB016) |
| Phage MH16con1 TERL | OQ633116 | SRR23699540 | 1818 | 39565 | terminase large subunit | partial | uncultured Caudovirales phage | [TPA: Large Terminase [Siphoviridae sp.]](https://blast.ncbi.nlm.nih.gov/Blast.cgi#alnHdr_DAY99226) | 98.51 | [DAY99226.1](https://www.ncbi.nlm.nih.gov/protein/DAY99226.1?report=genbank&log$=prottop&blast_rank=1&RID=Z45Y7YE5016) |
| Phage MH31con2 TERL | OQ633117 | SRR23699616 | 1518 | 88434 | terminase large subunit | partial | uncultured Caudovirales phage | [Terminase [Bacteriophage sp.]](https://blast.ncbi.nlm.nih.gov/Blast.cgi#alnHdr_UVX64103) | 99.8 | [UVX64103.1](https://www.ncbi.nlm.nih.gov/protein/UVX64103.1?report=genbank&log$=prottop&blast_rank=1&RID=Z45YHU1U013) |
| Phage MH35con3 TERL | OQ633118 | SRR23699646 | 1386 | 89958 | terminase large subunit | partial | uncultured Caudovirales phage | [TPA: large terminase [Siphoviridae sp.]](https://blast.ncbi.nlm.nih.gov/Blast.cgi#alnHdr_DAY94103) | 97.18 | [DAY94103.1](https://www.ncbi.nlm.nih.gov/protein/DAY94103.1?report=genbank&log$=prottop&blast_rank=5&RID=Z45YVE0701R) |
