# Supplementary material for: Viral metagenomics of the gut virome of diarrheal children with Rotavirus A infection
Source: Gut Microbes. 2023 Jul 13;15(1):2234653. doi: 10.1080/19490976.2023.2234653 (PMC10351451; doi:10.1080/19490976.2023.2234653)
Supplement: Supplemental Material [file KGMI_A_2234653_SM9927.zip › Supplemental material/Supplementary Table S4.docx]

**Supplementary Table S4**. Molecular epidemiology of enteric viruses in different samples

| Library | Sample | Rotavirus A | Adenovirus F | Astrovirus 1 | Astrovirus 5 | Astrovirus MLB1 | Norovirus GⅡ.2 | Norovirus GⅡ.3 | Norovirus GⅡ.4 | Coxsackievirus B5-1 | Coxsackievirus B5-2 | Coxsackievirus B2 | Coxsackievirus A4 |
| --- | --- | --- | --- | --- | --- | --- | --- | --- | --- | --- | --- | --- | --- |
| MH01 | 1X7M | positive | negative | positive | negative | negative | positive | negative | negative | negative | negative | positive | negative |
|  | 2X3Y | positive | negative | positive | negative | negative | negative | negative | negative | positive | negative | negative | negative |
|  | 3X7M | positive | negative | positive | negative | negative | negative | negative | negative | positive | negative | negative | negative |
|  | 4X5Y | positive | negative | negative | negative | negative | negative | negative | negative | negative | negative | negative | negative |
|  | 5M9M | positive | negative | negative | negative | negative | negative | negative | negative | negative | negative | negative | negative |
| MH02 | 6M4Y | positive | negative | negative | negative | negative | negative | negative | negative | negative | negative | negative | negative |
|  | 7X2Y | positive | negative | negative | negative | negative | positive | positive | negative | positive | negative | negative | positive |
|  | 8X2Y | positive | negative | negative | negative | negative | negative | negative | negative | negative | negative | positive | positive |
|  | 9X7Y | positive | negative | positive | negative | negative | negative | negative | negative | positive | negative | negative | negative |
|  | 10X3Y | positive | negative | negative | positive | negative | negative | negative | negative | negative | negative | negative | positive |
| MH03 | 11X9M | positive | negative | positive | negative | positive | positive | negative | positive | negative | negative | negative | negative |
|  | 12X2Y | positive | negative | positive | negative | positive | positive | negative | positive | negative | negative | negative | negative |
|  | 13X6Y | positive | negative | positive | negative | positive | negative | positive | negative | negative | negative | negative | negative |
|  | 14M5Y | positive | negative | negative | negative | positive | negative | negative | negative | negative | negative | positive | negative |
|  | 15X1Y | positive | negative | negative | negative | positive | positive | negative | negative | negative | positive | negative | negative |
| MH04 | 16F2Y | positive | negative | negative | negative | negative | positive | positive | negative | negative | negative | negative | negative |
|  | 17X3Y | positive | negative | positive | positive | positive | positive | negative | negative | negative | negative | negative | positive |
|  | 18X6Y | positive | negative | negative | negative | negative | positive | positive | negative | positive | negative | positive | negative |
|  | 19X6Y | negative | negative | negative | negative | negative | negative | negative | negative | negative | negative | negative | negative |
|  | 20X4Y | positive | negative | negative | negative | negative | negative | negative | negative | negative | negative | positive | negative |
| MH05 | 21M2Y | positive | negative | negative | negative | negative | negative | negative | positive | negative | negative | negative | negative |
|  | 22X2Y | positive | negative | negative | negative | negative | negative | negative | negative | negative | negative | negative | negative |
|  | 23X2Y | positive | negative | positive | negative | negative | negative | negative | negative | negative | positive | negative | negative |
|  | 24X4M | positive | negative | negative | negative | negative | negative | negative | negative | negative | negative | negative | positive |
|  | 25X2Y | positive | negative | positive | positive | negative | positive | negative | negative | negative | negative | negative | negative |
| MH06 | 26X2Y | positive | negative | negative | negative | negative | negative | negative | positive | negative | negative | negative | negative |
|  | 27X7Y | positive | positive | negative | negative | positive | negative | negative | negative | negative | negative | positive | negative |
|  | 28F3Y | negative | negative | negative | negative | negative | negative | negative | negative | positive | negative | negative | negative |
|  | 29M4Y | positive | negative | positive | positive | negative | positive | negative | negative | negative | negative | negative | negative |
|  | 30X6Y | positive | positive | negative | negative | negative | negative | negative | negative | positive | negative | negative | negative |
| MH07 | 31X1.5Y | positive | positive | negative | negative | positive | negative | positive | negative | negative | positive | negative | negative |
|  | 32X4Y | positive | negative | negative | negative | negative | negative | negative | positive | negative | negative | negative | negative |
|  | 33X2Y | positive | negative | negative | negative | negative | negative | negative | negative | negative | positive | positive | negative |
|  | 34F2Y5M | positive | negative | negative | negative | negative | negative | negative | negative | negative | negative | negative | positive |
|  | 35X2Y | positive | negative | negative | negative | negative | negative | negative | negative | negative | negative | negative | negative |
| MH08 | 36M5M | positive | positive | negative | negative | negative | negative | negative | negative | negative | negative | negative | negative |
|  | 37X28D | positive | positive | negative | negative | negative | negative | negative | positive | negative | negative | negative | negative |
|  | 38X7Y | positive | negative | negative | negative | positive | negative | negative | positive | negative | negative | negative | negative |
|  | 39X4Y | positive | positive | positive | negative | negative | positive | negative | negative | negative | negative | positive | positive |
|  | 40F4M | positive | negative | negative | negative | negative | negative | negative | negative | negative | negative | negative | negative |
| MH09 | 41M10M | positive | positive | negative | negative | negative | negative | positive | negative | positive | negative | negative | negative |
|  | 42M5Y | positive | positive | negative | negative | positive | negative | negative | negative | negative | negative | negative | positive |
|  | 43X45D | positive | positive | negative | negative | negative | negative | negative | negative | positive | negative | positive | negative |
|  | 44X2Y | positive | negative | positive | negative | negative | negative | negative | positive | negative | negative | negative | negative |
|  | 45X2Y | positive | negative | negative | negative | negative | positive | negative | negative | positive | negative | negative | negative |
| MH10 | 46X2Y | positive | negative | positive | negative | negative | negative | negative | negative | negative | negative | negative | negative |
|  | 47X2Y | positive | negative | negative | negative | positive | negative | negative | negative | negative | negative | negative | negative |
|  | 48X2Y | positive | negative | negative | negative | negative | negative | negative | negative | positive | negative | negative | negative |
|  | 49X8Y | positive | positive | negative | negative | negative | positive | negative | negative | negative | negative | negative | negative |
|  | 50X4M | positive | negative | positive | negative | positive | negative | negative | negative | negative | negative | positive | negative |
| MH11 | 51X5Y | positive | negative | negative | negative | negative | negative | negative | negative | positive | negative | negative | negative |
|  | 52X2Y | positive | negative | negative | negative | negative | negative | negative | negative | negative | negative | negative | positive |
|  | 53X6M | positive | negative | negative | negative | negative | negative | negative | negative | negative | negative | negative | negative |
|  | 54F20M | positive | negative | negative | negative | positive | negative | positive | negative | negative | negative | negative | negative |
|  | 55X3Y | positive | negative | negative | negative | negative | negative | negative | negative | negative | negative | negative | negative |
| MH12 | 56X3Y | positive | positive | negative | negative | negative | negative | negative | negative | negative | negative | positive | negative |
|  | 57M2Y | positive | negative | positive | negative | negative | negative | negative | negative | negative | negative | negative | negative |
|  | 58M5M | positive | negative | positive | negative | positive | negative | negative | negative | negative | negative | negative | negative |
|  | 59M10M | positive | negative | positive | negative | negative | negative | negative | negative | positive | negative | negative | positive |
|  | 60X8M | positive | negative | negative | negative | negative | negative | negative | negative | negative | negative | negative | negative |
| MH13 | 61F1.5Y | positive | negative | negative | negative | negative | negative | negative | negative | negative | negative | positive | negative |
|  | 62F22M | positive | negative | positive | negative | negative | negative | positive | positive | negative | negative | negative | negative |
|  | 63M3Y | positive | negative | negative | negative | positive | negative | negative | positive | positive | negative | negative | negative |
|  | 64F3Y | positive | negative | negative | negative | negative | negative | negative | negative | negative | negative | positive | negative |
|  | 65M10Y | positive | negative | positive | negative | negative | negative | negative | negative | negative | negative | negative | negative |
| MH14 | 66F10M | positive | negative | positive | negative | positive | negative | negative | negative | negative | negative | positive | negative |
|  | 67M1D | positive | negative | positive | negative | negative | negative | negative | negative | positive | negative | negative | negative |
|  | 68M5Y | positive | negative | negative | negative | negative | positive | negative | negative | negative | positive | negative | positive |
|  | 69F1Y | positive | negative | positive | negative | negative | negative | negative | negative | negative | negative | negative | negative |
|  | 70M14M | positive | negative | negative | negative | negative | negative | negative | negative | positive | negative | negative | negative |
| MH15 | 71F9M | positive | negative | negative | negative | positive | negative | negative | negative | negative | negative | negative | negative |
|  | 72F13M | positive | negative | negative | negative | negative | negative | negative | positive | negative | positive | positive | negative |
|  | 73M6Y | positive | negative | negative | negative | negative | negative | negative | negative | positive | negative | negative | negative |
|  | 74F16M | positive | negative | negative | negative | negative | negative | negative | negative | negative | positive | negative | positive |
|  | 75M1Y | positive | negative | negative | negative | negative | positive | positive | negative | negative | negative | negative | negative |
|  | 76M2Y | positive | negative | negative | negative | positive | negative | negative | positive | negative | negative | negative | negative |
| MH16 | TIF11Y | positive | negative | negative | negative | negative | negative | negative | negative | negative | negative | positive | negative |
|  | T2F1Y | positive | negative | positive | positive | negative | negative | negative | negative | negative | negative | negative | negative |
|  | T3M1Y | positive | negative | negative | negative | negative | negative | negative | negative | negative | negative | negative | positive |
|  | T4M1Y | positive | negative | negative | negative | negative | negative | positive | negative | positive | positive | negative | negative |
|  | T5M1Y | positive | negative | negative | negative | negative | negative | negative | negative | negative | positive | positive | negative |
| MH17 | T6M1Y | positive | negative | negative | negative | negative | negative | negative | negative | negative | negative | negative | positive |
|  | T7F4M | positive | negative | negative | positive | negative | negative | negative | negative | positive | negative | negative | negative |
|  | T8XX | positive | negative | negative | negative | positive | negative | negative | negative | negative | negative | negative | negative |
|  | T9M5Y | positive | negative | negative | negative | negative | negative | positive | negative | negative | negative | positive | positive |
|  | T10M3Y | positive | negative | positive | positive | negative | negative | negative | negative | positive | positive | negative | negative |
| MH18 | T11F11Y | positive | negative | negative | negative | positive | negative | positive | negative | negative | negative | negative | negative |
|  | T12M1Y | positive | negative | negative | negative | negative | negative | negative | positive | negative | negative | positive | negative |
|  | T13F3M | positive | negative | positive | negative | negative | negative | negative | negative | negative | negative | negative | negative |
|  | T14M1Y | positive | negative | negative | positive | positive | negative | positive | negative | negative | negative | negative | negative |
|  | T15F9M | positive | negative | negative | negative | negative | negative | negative | negative | negative | negative | positive | positive |
| MH19 | T16M7M | positive | negative | negative | negative | negative | negative | negative | negative | negative | negative | negative | negative |
|  | T17F7Y | positive | negative | positive | negative | positive | negative | negative | negative | negative | positive | negative | negative |
|  | T18M5M | positive | negative | negative | negative | negative | negative | negative | negative | negative | positive | negative | negative |
|  | T19F1Y | positive | negative | negative | negative | negative | negative | negative | negative | negative | negative | positive | negative |
|  | T20F4Y | positive | negative | positive | negative | negative | negative | positive | negative | negative | positive | negative | negative |
| MH20 | T21F3Y | positive | positive | negative | negative | negative | negative | negative | negative | negative | negative | negative | negative |
|  | T22XX | positive | negative | negative | negative | positive | negative | positive | negative | negative | negative | negative | negative |
|  | T23M1Y | positive | positive | negative | positive | negative | negative | negative | positive | negative | positive | negative | negative |
|  | T24M9M | positive | negative | negative | negative | negative | negative | negative | negative | negative | negative | positive | positive |
|  | T25XX | positive | negative | negative | negative | positive | negative | negative | negative | negative | negative | negative | negative |
| MH21 | T26XX | positive | negative | negative | negative | negative | negative | negative | positive | negative | negative | negative | negative |
|  | T27F6M | positive | positive | negative | negative | negative | negative | negative | negative | negative | negative | negative | negative |
|  | T28M2Y | positive | negative | negative | negative | negative | negative | positive | negative | negative | negative | negative | negative |
|  | T29M1Y | positive | negative | positive | negative | positive | negative | negative | negative | negative | negative | positive | positive |
|  | T30XX | positive | negative | negative | negative | negative | negative | positive | negative | negative | negative | negative | negative |
| MH22 | T31F1Y | positive | negative | negative | positive | negative | negative | negative | negative | negative | positive | negative | negative |
|  | T32XX | positive | negative | negative | negative | negative | negative | negative | negative | negative | negative | positive | negative |
|  | T33M3Y | positive | negative | negative | negative | negative | negative | negative | negative | negative | negative | negative | negative |
|  | T34XX | positive | negative | negative | negative | negative | negative | negative | negative | negative | negative | negative | negative |
|  | T35XX | positive | negative | negative | negative | negative | negative | positive | negative | negative | positive | negative | negative |
| MH24 | T41M4M | positive | negative | positive | positive | negative | negative | negative | positive | negative | negative | negative | negative |
|  | T42F7Y | positive | negative | negative | negative | positive | negative | negative | negative | negative | negative | positive | negative |
|  | T43XX | positive | negative | negative | negative | negative | negative | negative | negative | positive | negative | negative | negative |
|  | T44M13Y | positive | negative | negative | negative | negative | negative | positive | negative | negative | negative | negative | negative |
|  | T45XX | positive | negative | negative | negative | negative | negative | negative | negative | positive | negative | positive | negative |
| MH28 | T1H | negative | positive | negative | negative | negative | negative | negative | positive | negative | negative | positive | negative |
|  | T2H | negative | negative | negative | negative | negative | negative | negative | negative | negative | positive | positive | negative |
|  | T3H | negative | negative | positive | negative | negative | negative | negative | negative | negative | positive | positive | negative |
|  | T4H | negative | negative | negative | negative | negative | positive | negative | negative | negative | negative | positive | negative |
|  | T5H | negative | negative | negative | negative | negative | negative | negative | positive | negative | negative | negative | negative |
| MH29 | T6H | negative | negative | positive | negative | negative | negative | negative | negative | negative | negative | negative | negative |
|  | T7H | negative | negative | negative | negative | negative | negative | negative | positive | negative | negative | positive | negative |
|  | T8H | negative | negative | negative | negative | negative | positive | negative | positive | positive | negative | positive | negative |
|  | T9H | negative | negative | negative | negative | negative | negative | negative | negative | negative | negative | negative | negative |
|  | T10H | negative | negative | positive | negative | negative | positive | negative | negative | positive | positive | positive | negative |
| MH30 | T11H | negative | negative | negative | negative | negative | negative | negative | positive | negative | negative | negative | negative |
|  | T12H | negative | negative | negative | negative | negative | negative | negative | negative | negative | negative | negative | negative |
|  | T13H | negative | negative | negative | negative | positive | negative | negative | positive | negative | negative | negative | negative |
|  | T14H | positive | positive | negative | negative | negative | negative | negative | negative | negative | negative | negative | negative |
|  | T15H | negative | negative | negative | negative | negative | negative | negative | negative | negative | negative | negative | negative |
| MH31 | T16H | negative | negative | negative | negative | negative | negative | negative | negative | negative | negative | negative | negative |
|  | T17H | positive | negative | negative | negative | negative | negative | negative | negative | negative | negative | negative | negative |
|  | T18H | positive | negative | negative | negative | negative | negative | negative | negative | negative | negative | negative | negative |
|  | T19H | positive | negative | negative | negative | negative | negative | negative | negative | negative | negative | negative | negative |
| MH32 | 1HH | negative | negative | negative | negative | negative | negative | negative | negative | negative | negative | negative | negative |
|  | 2HH | negative | negative | negative | negative | negative | positive | positive | positive | positive | negative | negative | negative |
|  | 3HH | negative | negative | negative | negative | negative | negative | negative | negative | negative | negative | negative | negative |
|  | 4HH | negative | negative | negative | negative | positive | negative | negative | positive | negative | positive | negative | negative |
|  | 5HH | negative | negative | positive | negative | negative | negative | negative | positive | negative | negative | negative | negative |
| MH33 | 6HH | negative | negative | negative | negative | negative | positive | negative | negative | positive | negative | negative | negative |
|  | 7HH | negative | negative | negative | negative | negative | positive | negative | negative | positive | negative | negative | positive |
|  | 8HH | negative | negative | negative | negative | negative | negative | negative | negative | negative | negative | negative | negative |
|  | 9HH | negative | negative | negative | negative | positive | positive | negative | positive | positive | negative | negative | negative |
|  | 10HH | negative | negative | positive | negative | negative | negative | negative | positive | negative | positive | negative | negative |
| MH34 | 11HH | negative | negative | negative | negative | negative | positive | negative | positive | positive | positive | negative | negative |
|  | 12HH | negative | negative | positive | negative | negative | negative | negative | positive | negative | negative | negative | negative |
|  | 13HH | positive | positive | negative | negative | positive | negative | negative | positive | positive | negative | negative | negative |
|  | 14HH | positive | negative | negative | negative | negative | negative | negative | negative | negative | negative | negative | negative |
|  | 15HH | negative | negative | negative | negative | negative | positive | negative | positive | negative | negative | negative | negative |
| MH35 | 16HH | negative | negative | negative | negative | negative | negative | negative | negative | negative | negative | negative | negative |
|  | 17HH | negative | negative | negative | negative | negative | negative | negative | negative | positive | negative | negative | negative |
|  | 18HH | negative | negative | negative | negative | negative | positive | negative | positive | positive | negative | positive | negative |
|  | 19HH | negative | negative | negative | negative | positive | positive | negative | negative | negative | negative | negative | negative |
|  | 20HH | negative | negative | negative | negative | negative | negative | negative | negative | negative | negative | negative | negative |
| MH36 | 21HH | negative | negative | positive | negative | negative | negative | positive | positive | positive | negative | negative | negative |
|  | 22HH | negative | negative | negative | negative | negative | positive | negative | negative | negative | negative | negative | negative |
|  | 23HH | negative | negative | negative | positive | negative | positive | negative | negative | positive | negative | positive | negative |
|  | 24HH | negative | negative | negative | negative | negative | positive | negative | negative | positive | negative | negative | negative |
|  | 25HH | negative | negative | negative | negative | negative | negative | negative | negative | negative | negative | positive | negative |
| MH37 | 26HH | negative | negative | negative | negative | negative | positive | negative | positive | positive | negative | negative | negative |
|  | 27HH | negative | negative | negative | negative | negative | negative | positive | positive | negative | negative | positive | negative |
|  | 28HH | negative | negative | negative | negative | negative | negative | negative | positive | negative | negative | negative | negative |
|  | 29HH | negative | negative | negative | negative | negative | negative | negative | negative | positive | negative | negative | negative |
|  | 30HH | negative | negative | negative | negative | negative | positive | negative | positive | negative | negative | positive | negative |
|  | 31HH | negative | negative | negative | negative | negative | positive | negative | negative | positive | negative | negative | negative |
|  | 32HH | negative | negative | negative | negative | negative | positive | negative | negative | negative | negative | positive | negative |
